# Bile-induced biofilm formation in *Bacteroides thetaiotaomicron* requires magnesium efflux by an RND pump

**DOI:** 10.1128/mbio.03488-23

**Published:** 2024-03-27

**Authors:** Anne-Aurélie Lopes, Sol Vendrell-Fernández, Julien Deschamps, Sonia Georgeault, Thomas Cokelaer, Romain Briandet, Jean-Marc Ghigo

**Affiliations:** 1 Institut Pasteur, Université Paris-Cité, UMR CNRS 6047, Genetics of Biofilms Laboratory, Department of Microbiology, Paris, France; 2 Pediatric Emergency, AP-HP, Necker-Enfants-Malades University Hospital, Paris, France; 3 INRAE, AgroParisTech, Université Paris-Saclay Institut Micalis, Paris, France; 4 Plateforme IBiSA des Microscopies, Université et CHRU de Tours, Tours, France; 5 Institut Pasteur, Université Paris Cité, Plate-forme Technologique Biomics, Center for Technological Resources and Research, Paris, France; 6 Institut Pasteur, Université Paris Cité, Bioinformatics and Biostatistics Hub, Center for Technological Resources and Research, Paris, France; University of Washington, Seattle, Washington, USA

**Keywords:** *Bacteroides thetaiotaomicron*, bile salts, biofilm matrix, RND-efflux pumps, eDNA

## Abstract

*Bacteroides thetaiotaomicron* is a prominent member of the human gut microbiota contributing to nutrient exchange, gut function, and maturation of the host’s immune system. This obligate anaerobe symbiont can adopt a biofilm lifestyle, and it was recently shown that *B. thetaiotaomicron* biofilm formation is promoted by the presence of bile. This process also requires a *B. thetaiotaomicron* extracellular DNase, which is not, however, regulated by bile. Here, we showed that bile induces the expression of several Resistance-Nodulation-Division (RND) efflux pumps and that inhibiting their activity with a global competitive efflux inhibitor impaired bile-dependent biofilm formation. We then showed that, among the bile-induced RND-efflux pumps, only the tripartite BT3337-BT3338-BT3339 pump, re-named BipABC [for Bile Induced Pump A (*BT3337*), B (*BT3338*), and C (*BT3339*)], is required for biofilm formation. We demonstrated that BipABC is involved in the efflux of magnesium to the biofilm extracellular matrix, which leads to a decrease of extracellular DNA concentration. The release of magnesium in the biofilm matrix also impacts biofilm structure, potentially by modifying the electrostatic repulsion forces within the matrix, reducing interbacterial distance and allowing bacteria to interact more closely and form denser biofilms. Our study therefore, identified a new molecular determinant of *B. thetaiotaomicron* biofilm formation in response to bile salts and provides a better understanding on how an intestinal chemical cue regulates biofilm formation in a major gut symbiont.

**IMPORTANCE:**

*Bacteroides thetaiotaomicron* is a prominent member of the human gut microbiota able to degrade dietary and host polysaccharides, altogether contributing to nutrient exchange, gut function, and maturation of the host’s immune system. This obligate anaerobe symbiont can adopt a biofilm community lifestyle, providing protection against environmental factors that might, in turn, protect the host from dysbiosis and dysbiosis-related diseases. It was recently shown that *B. thetaiotaomicron* exposure to intestinal bile promotes biofilm formation. Here, we reveal that a specific *B. thetaiotaomicron* membrane efflux pump is induced in response to bile, leading to the release of magnesium ions, potentially reducing electrostatic repulsion forces between components of the biofilm matrix. This leads to a reduction of interbacterial distance and strengthens the biofilm structure. Our study, therefore, provides a better understanding of how bile promotes biofilm formation in a major gut symbiont, potentially promoting microbiota resilience to stress and dysbiosis events.

## INTRODUCTION

*Bacteroides thetaiotaomicron* is a prominent human gut symbiont involved in beneficial nutrient exchanges, gut functions, and maturation of the host’s immune system from the earliest stages of life (1–5). *B. thetaiotaomicron* can degrade dietary and host polysaccharides and is often found adhering to food particles or grazing on the polysaccharide-rich gut mucus, developing biofilm-like communities that provide a stable and protective environment that also favors genetic exchange (2, 6–8). Several factors promoting the formation of *B. thetaiotaomicron* biofilm have been identified, including polysaccharide utilization receptors, capsular polysaccharides, and type V pili (9–12). We also recently showed that physiological concentrations of bile salts induce biofilm formation in a wide range of *B. thetaiotaomicron* strains and many Bacteroidales, suggesting that this relevant gut compound, beyond its critical digestive roles, could also induce biofilm formation *in vivo* (13, 14). Whereas bile-dependent biofilm formation requires the BT3563 DNase activity degrading extracellular DNA (eDNA) (14), the expression of *BT3563* gene is not induced by bile salts, and the link between bile exposure and biofilm formation is, therefore, still unclear.

We and others previously showed that bile salts induce the expression of tripartite Resistance-Nodulation-Division (RND) efflux pumps in various bacteria, including *B. thetaiotaomicron* (14–18). Whereas this process could contribute to reduce bile toxicity, RND-efflux pumps also actively transport a broad range of substrates (4, 17, 19–21). Here, we hypothesized that *B. thetaiotaomicron* RND-type efflux pumps could directly contribute to bile-dependent biofilm formation by secreting compounds into the biofilm matrix. We first showed that blocking RND-efflux pumps with the efflux inhibitor phenylalanine-arginine beta-naphthylamide (PAßN) reduced biofilm formation. By comparing gene expression in the presence and absence of bile, we determined that bile induces the expression of seven *B. thetaiotaomicron* RND-efflux pumps, and we showed that only the tripartite efflux pump encoded by the *BT3337*, *BT3338*, and *BT3339* gene cluster is required for bile-dependent biofilm formation. We then demonstrated that this pump, renamed BipABC, exports magnesium divalent cations, which decreases the concentration of eDNA in the *B. thetaiotaomicron* extracellular matrix, allowing bacteria to interact more closely and form more compact biofilm structures. The demonstration of a direct link between exposure to bile and the expression of a specific RND-type pump and magnesium efflux provides new insights into the regulation of biofilm formation in *B. thetaiotaomicron*.

## RESULTS

### Inhibiting *B. thetaiotaomicron* RND-efflux pumps impairs bile-dependent biofilm formation

**Fig 1:**
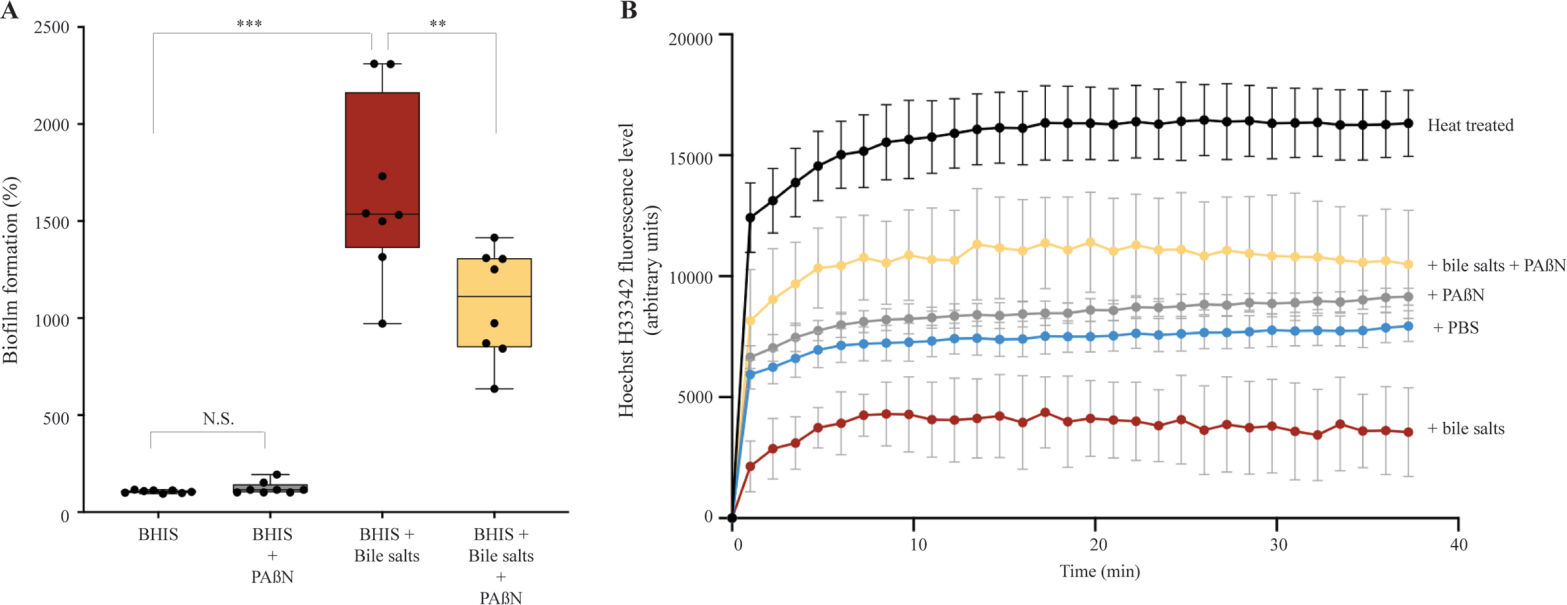
RND-type efflux is involved in bile-dependent biofilm formation. (A) Ninety-six-well plate crystal violet assay of *B. thetaiotaomicron* VPI-5482 biofilm formation after 48 h growth in Brain heart infusion medium (BHIS in the absence and presence of PAßN (25 µg/mL) and/or 0.5% bile salts (BS). Mean of wild-type biofilm formation in BHIS was adjusted to 100%. Min-max boxplot of eight biological replicates, each of them being the mean of three technical replicates, for each condition. NS: non-significant. ** *P*-value < 0.005; *** *P*-value < 0.0005. Statistics correspond to an unpaired, nonparametric Mann–Whitney *U* test. (**B**) Hoechst H33342 accumulation assay (2.5 µM) in *B. thetaiotaomicron* VPI-5482 in the absence and presence of 0.5% BS and/or PAßN (25 µg/mL). Hoechst H33342 level in heat-inactivated bacteria represents the maximum fluorescence level. Mean of four biological replicates.

To evaluate the contribution of RND-efflux pumps to *B. thetaiotaomicron* VPI-5482 biofilm formation in the presence of bile salts, we tested the impact of blocking its RND-type efflux pumps with the competitive efflux pump inhibitor PAßN (22, 23). We observed that the addition of 25 µg/mL PAßN significantly inhibited bile-dependent biofilm formation (Fig. 1A), while the addition of PAßN did not affect growth or viability in planktonic culture and in biofilm conditions (Fig. S1A through C). We also verified that PAßN did not increase membrane permeability since exposure to PAßN did not modify *B. thetaiotaomicron* VPI-5482 sensitivity to vancomycin, an antibiotic unable to cross the outer membrane of Gram-negative bacteria unless permeabilized (Table S1) (24). Finally, we used Hoechst H33342 as a probe for efflux activity in a real-time drug accumulation assay (25) to demonstrate the specific impact of PAßN on efflux. We first showed that, compared to the phosphate-buffered saline (PBS) control, the presence of bile decreased the intracellular concentration of Hoechst H33342, indicative of its increased efflux (Fig. 1B). By contrast, dual exposure to bile and PAßN increased the intracellular accumulation of Hoechst H33342, indicating that PAßN blocked bile-induced RND-type efflux (Fig. 1B). Taken together, these results indicated that *B. thetaiotaomicron* VPI-5482 RND-type efflux contributes to bile-dependent biofilm formation.

### The RND-efflux pump BT3337-3339 is required for bile-dependent biofilm formation

**Fig 2:**
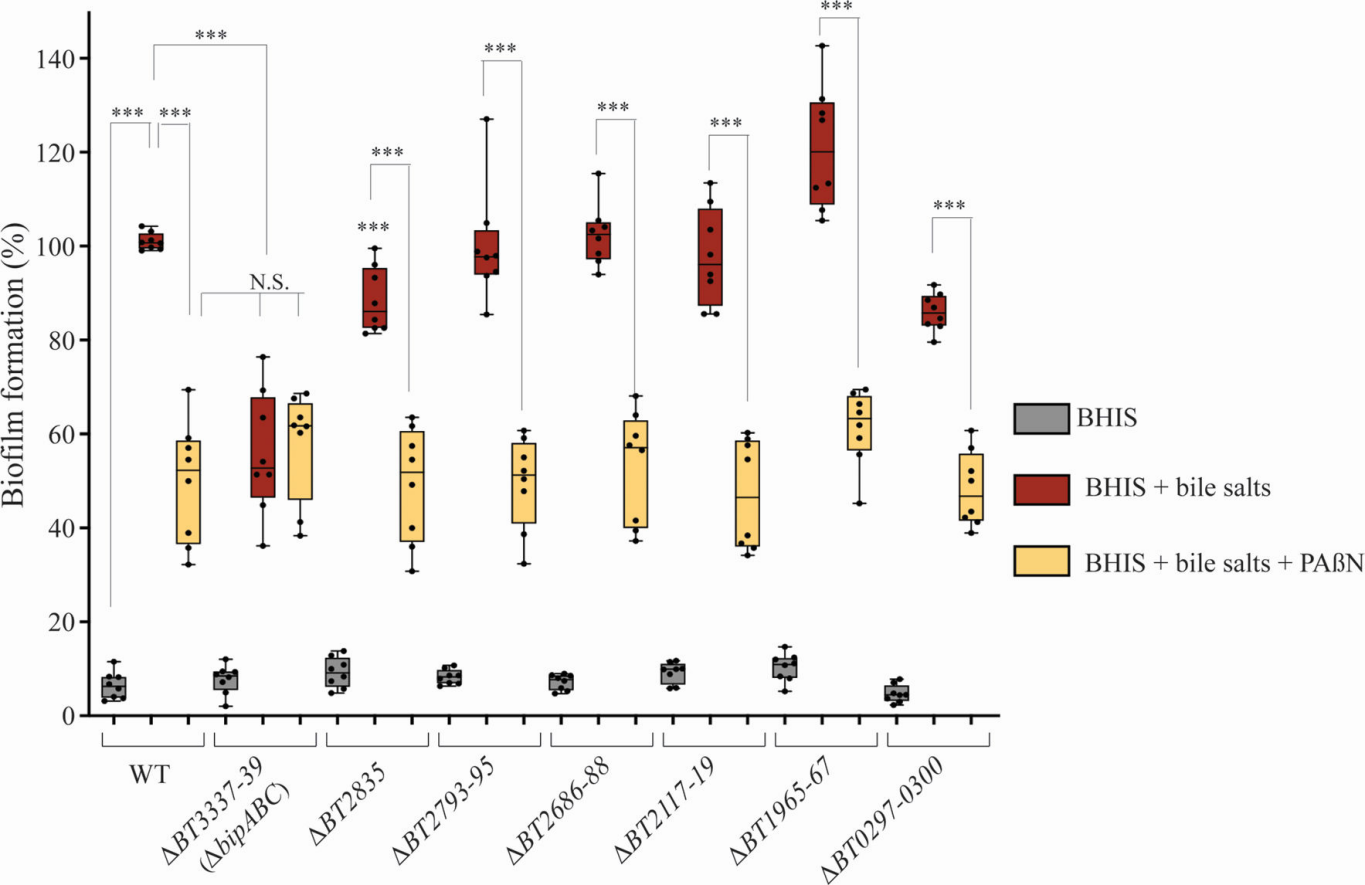
The deletion of *BT3337-39* gene cluster reduced *B. thetaiotaomicron* VPI-5482 biofilm formation. Ninety-six-well plate crystal violet biofilm assay after 48 h growth in BHIS in the absence and presence of 0.5% bile salts without or with 25 µg/mL of PAßN of strains in which each of the seven-targeted RND-type efflux pumps clusters were deleted. Mean of WT in BHIS with 0.5% BS was adjusted to 100%. Min-max boxplot of eight biological replicates, each of them being the mean of six technical replicates, for each condition. *** *P*-value < 0.0005; NS: non-significant compared to WT in BHIS with BS. Statistics correspond to an unpaired, nonparametric Mann–Whitney *U* test.

To identify which specific *B. thetaiotaomicron* efflux pumps could be involved in biofilm formation, we performed an RNA-seq analysis to compare the expression of the 21 gene clusters annotated as RND-type efflux pumps in the VPI-5482 genome in the absence or presence of bile (26). This analysis confirmed the previously observed induction of the tripartite BT2793-2795 efflux pump (14), along with six other RND-efflux pumps clusters, which were induced close to or above a factor of 2 log2 in bile salt conditions (namely *BT0297-0300*, *BT1965-1967*, *BT2117-2119*, *BT2686-2688*, *BT2835*, and *BT3337-3339*; Table S2 and S3). We then deleted each of these seven RND-type efflux pump operons and showed that only the deletion of the *BT3337-3339* gene cluster led to a reduction of biofilm capacity to a level similar to the one obtained upon the addition of PAβN in the wild-type (WT) strain (Fig. 2). This biofilm defect could be complemented by providing the *BT3337-3339* gene cluster in *trans* (Fig. S2). Moreover, the addition of PAβN did not further reduce biofilm formation of the ∆*BT3337-3339* mutant (Fig. 2). All other RND-type efflux pump mutants either only mildly decreased or even increased (*BT1965-67*) biofilm formation, while the addition of PAßN still significantly decreases the biofilm formation (Fig. 2). These results indicated the involvement of the BT3337-3339 RND-type efflux pump in biofilm formation in the presence of bile, and this pump was hereafter renamed BipABC, for Bile-Induced Pump A (*BT3337*), B (*BT3338*), and C (*BT3339*).

### The *B. thetaiotaomicron* eDNA content increases in the absence of the BipABC efflux pump

**Fig 3:**
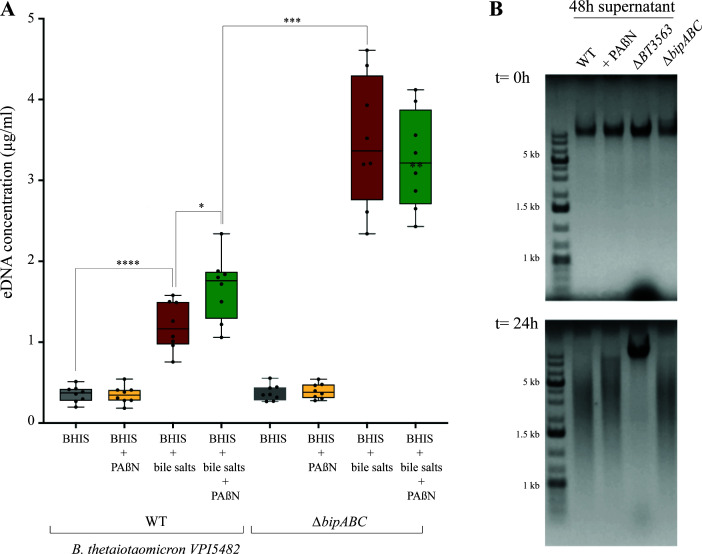
The addition of PAßN and deletion of *bipABC* (*BT3337-39)* genes lead to eDNA increase without impairing the BT3563 extracellular DNase activity. (A) The concentration of DNA in the extracellular matrix of *B. thetaiotaomicron* VPI-5482 WT or its corresponding ∆*bipABC* mutant with 0.5% bile salts (BS) and 25 µg/mL of PAßN as indicated. Min-max boxplot of eight biological replicates for each condition. * *P*-value < 0.05; *** *P*-value < 0.0005. Statistics correspond to an unpaired, nonparametric Mann–Whitney *U* test. (**B)** Incubation of *B. thetaiotaomicron* genomic DNA (800 ng/mL final concentration) with the supernatant of 48-h bile-free indicated cultures and loaded immediately on a 1% agarose gel (*t* = 0 h) and after 24 h incubation (*t* = 24 h) at 37°C.

To investigate how *B. thetaiotaomicron* efflux pumps contributed to bile-dependent biofilm formation, we compared the amount of eDNA, proteins, and polysaccharides in the extracellular matrix of biofilms formed in the absence and presence of PAßN. After normalization to the extent of recovered biomass, we observed that the addition of bile salts increased the concentrations of all three matrix components (Fig. 3A; Fig. S3). By contrast, compared to the addition of bile salts alone, the addition of bile salts and PAßN led to an increase in eDNA, which was also observed in the matrix extracted from a *∆bipABC* mutant (Fig. 3A). The addition of PAßN to bile salts did not change the concentration of eDNA in the matrix of the ∆bipABC mutant (Fig. 3A). Moreover, the number of bacteria by milligram of extracellular matrix was similar in the WT and in the *∆bipABC* mutant, in all tested conditions (Fig. S1B and C). To test whether this eDNA increase could result from a reduction of eDNA degradation, we exposed for 24 h the *B. thetaiotaomicron* genomic DNA to bacterial-free filtered culture supernatants of VPI-5482 WT and its *∆bipABC* mutant with bile or bile and PAßN. As a control, we exposed genomic DNA to filtered culture supernatants of a *∆BT3563* mutant lacking the previously described BT3563 extracellular DNase (14), which did not lead to eDNA degradation (Fig. 3B). All other conditions showed WT level of *B. thetaiotaomicron* genomic DNA degradation (Fig. 3B), suggesting that the lack of the BipABC efflux pump did not reduce the level of DNase activity in the tested supernatants (Fig. 3B).

### Addition of magnesium restores *B. thetaiotaomicron* bile-dependent biofilm in the ∆*bipABC* efflux pump mutant

**Fig 4:**
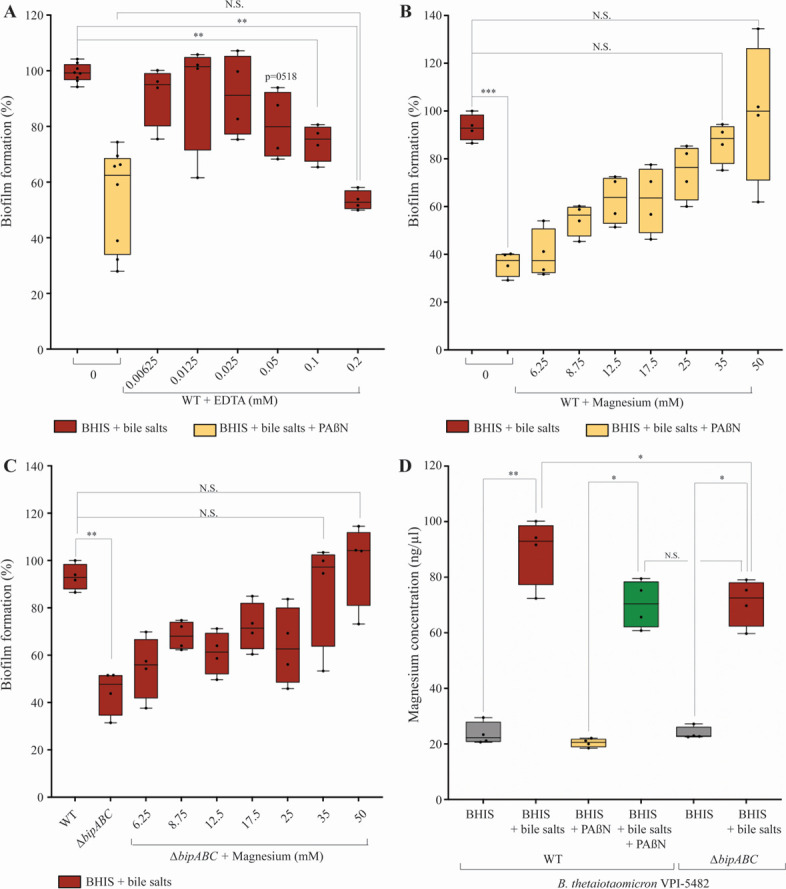
The addition of magnesium divalent ions restores RND-efflux pump defect in bile-dependent biofilm formation in *B. thetaiotaomicron*. (A and **B) **Ninety-six-well plate crystal violet biofilm assay after 48 h growth in BHIS of VPI-5482 in the presence of 0.5% bile salts without or with 25 µg/mL of PAßN and different non-toxic concentrations of EDTA (**A**) or magnesium (**B**). Mean of WT in BHIS with 0.5% BS was adjusted to 100%. Min-max boxplot of eight biological replicates for each condition. (**C)** Ninety-six-well plate crystal violet biofilm assay after 48 h growth in BHIS of ∆*bipABC* in the presence of 0.5% bile salts and different non-toxic concentrations of magnesium. Mean of WT in BHIS with 0.5% BS was adjusted to 100%. Min-max boxplot of eight biological replicates for each condition. (**D)** Quantification of magnesium concentration in the ECM in VPI-5482 without and with 0.5% bile salts or 25 µg/mL of PAßN and in ∆*bipABC* mutants without and with 0.5% BS. Min-max boxplot of eight biological replicates for each condition. * *P*-value < 0.05; ** *P*-value < 0.005; *** *P*-value < 0.0005; N.S.: non-significant. Statistics correspond to an unpaired, nonparametric Mann–Whitney *U* test.

Among potential substrates exported by RND-type efflux pumps, divalent cations have been reported to impact the interactions between biofilm matrix components (27, 28). To test whether impairing cation efflux could account for the biofilm defect observed in the ∆*bipABC* efflux pump mutant, we first evaluated the impact of EDTA, a chelator of a wide range of divalent cations, on bile-dependent biofilm formation. We showed that extracellular addition of EDTA inhibited bile-dependent biofilm formation reaching a level similar to PAßN inhibition at the non-toxic EDTA concentration of 0.2 mM (Fig. 4A; Fig. S3A). Among the cations potentially chelated by EDTA, we showed that the addition of non-toxic concentration of magnesium (35–50 mM) restored wild-type level of biofilm formation in the presence of PAßN (Fig. 4B; Fig. S3B). Consistently, the addition of magnesium also restored biofilm formation of the *∆bipABC* mutant (Fig. 4C; Fig. S3C). By contrast, such chemical complementation was not observed with the addition of calcium, cadmium, cobalt, iron, potassium, or zinc (Fig. S4A through F). Finally, we also confirmed that magnesium concentration was increased in the extracellular matrix of the WT strain *B. thetaiotaomicron* VPI-5482 grown in the presence of bile salts but decreased, compared to WT with bile salts, when grown in the presence of bile salts and PAßN (Fig. 4D). Compared to WT with bile salts, the magnesium concentration was also decreased in the extracellular matrix of the *∆bipABC* mutant in the presence of bile salts (Fig. 4D). These results further demonstrated that genetic inactivation or chemical inhibition of BipABC reduces magnesium-efflux and affects bile-dependent biofilm formation.

### Magnesium efflux decreases the concentration of extracellular DNA in *B. thetaiotaomicron* biofilm

**Fig 5:**
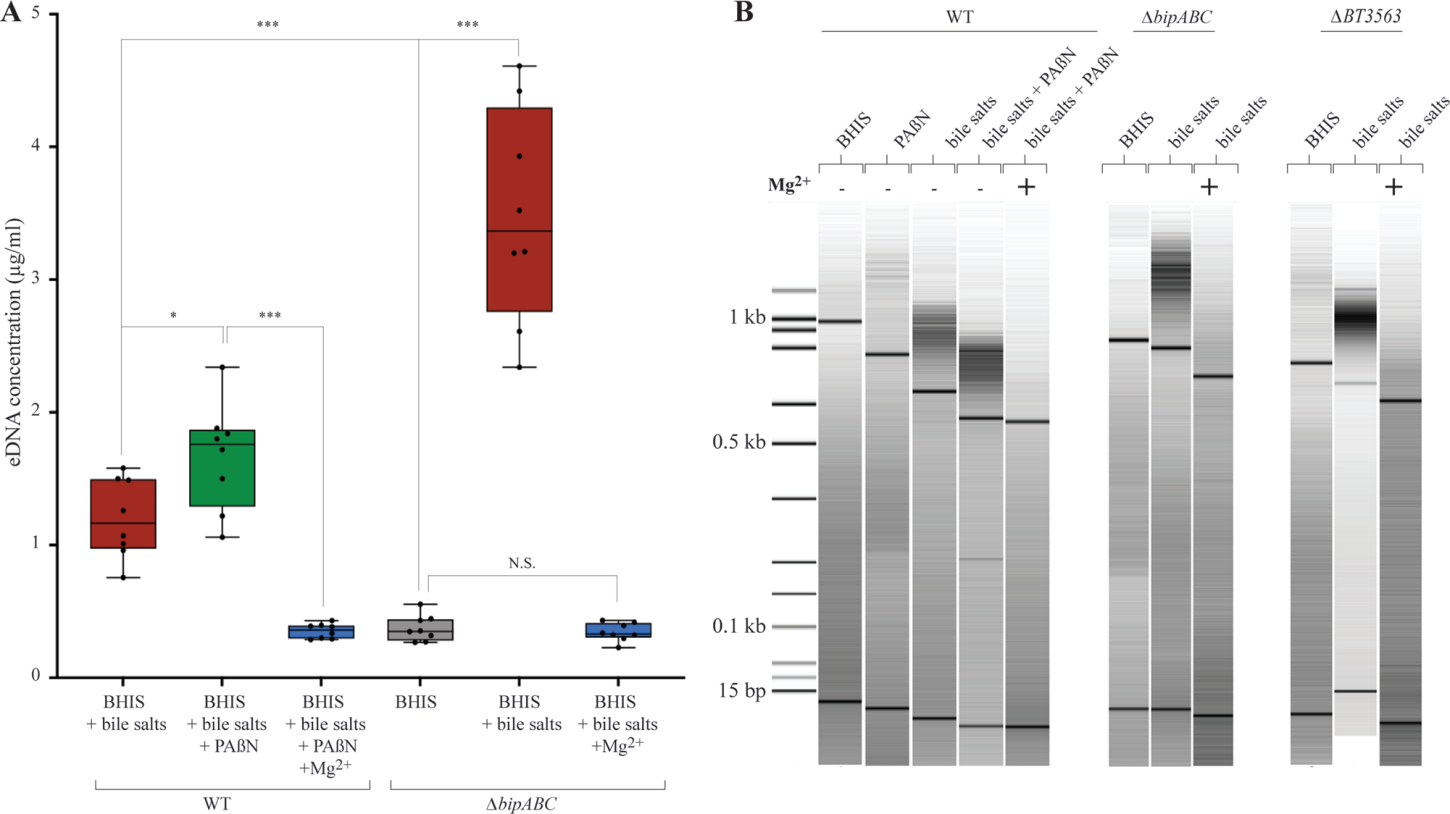
Quantification and effects of magnesium on eDNA concentration and profile. (**A)** The concentration of DNA in the extracellular matrix of VPI-5482 without and with 0.5% bile salts or 25 µg/mL of PAßN or 35 mM magnesium and in deleted mutants without and with 0.5% bile or 35 mM magnesium. Min-max boxplot of eight biological replicates for each condition. * *P*-value < 0.05 and *** *P*-value < 0.0005; N.S.: non-significant. Statistics correspond to an unpaired, nonparametric Mann–Whitney *U* test. (**B)** Bioanalyzer DNA profile of extracellular matrix extract samples.

In addition to restoring bile-dependent biofilm formation, we showed that extracellular addition of magnesium also reduced the concentration of eDNA in the extracellular matrix of WT *B. thetaiotaomicron* VPI-5482 grown in the presence of bile salts and PAßN as well as a *∆bipABC* mutant grown in the presence of bile salts (Fig. 5A). To investigate the effect of magnesium on eDNA, we use electrophoresis profiling to compare the size of eDNA fragments extracted from the extracellular matrix of biofilms formed by WT VPI-5482 and the ∆*bipABC* and ∆*BT3563* DNase mutant in the presence of bile salts with and without PAßN and magnesium. This analysis revealed that a high concentration of DNA fragments sized above 600 bp in the WT strains as well as in the mutants grown in the presence of bile (Fig. 5B). By contrast, the addition of magnesium eliminated this fraction above 600 bp eDNA fraction (Fig. 5B), suggesting that magnesium divalent cations could either activate eDNA degradation by other *B. thetaiotaomicron* DNases than BT3563 or limit the release of eDNA in the biofilm matrix.

### Magnesium efflux modifies biofilm structure by reducing interbacterial distance

**Fig 6:**
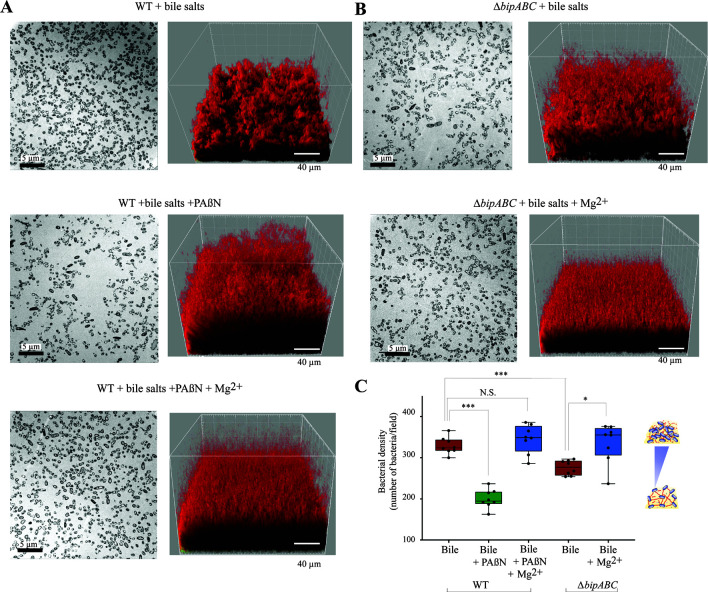
Increased interbacterial distance upon the addition of PAßN and deletion of the BipABC RND-type pump is complemented by the addition of magnesium. (A) Visualization of biofilms from *B. thetaiotaomicron* VPI-5482 WT. **(B)** Visualization of biofilms from the ∆*bipABC* deletion mutant. Left: TEM images of ultra-thin sections of resin-embedded biofilms (scale bar: 5 µm). Right: Imaris three-dimensional (3D) reconstruction of the biofilm from CLSM (scale bar: 40 µm). Bacterial cells are labeled in red with 5 µM of SYTO61, a cell permeant nucleic acids dye. Culture conditions correspond to 0.5% bile salts, 25 µg/mL of PAßN, or 35 mM magnesium. (**C)** Interbacterial distance was evaluated by the number of bacteria by observation field of ultra-thin section of TEM. Min-max boxplot of eight biological replicates for each condition. * *P*-value < 0.05; *** *P*-value < 0.0005; N.S.: non-significant. Statistics correspond to an unpaired, nonparametric Mann–Whitney *U* test.

To test the impact of magnesium efflux on the structure of *B. thetaiotaomicron* biofilm, we used transmission electron microscopy (TEM) performed on ultra-thin section of resin-embedded biofilms (29). We observed that, compared to WT, the inhibition of efflux with PAßN or the deletion of the ∆*bipABC* efflux pump genes led to a significant decrease of biofilm bacterial density correlating with an increased interbacterial distance (Fig. 6). This phenotype could be complemented by the addition of magnesium to VPI-5482 in the presence of PAßN or to the ∆*bipABC* mutant (Fig. 6). These results are consistent with the determination of biofilm biovolume after imaging of 48 h 96-well plate biofilms by confocal laser scanning microscopy (CLSM), showing that blocking all RND-efflux pump (PAßN condition), or only BipABC in the presence of bile increased biovolume, which was corrected upon the addition of magnesium (Fig. S5). These results demonstrate the key role played by magnesium efflux and the bile-dependent BipABC efflux pump in the formation of dense biofilm in the presence of bile.

## DISCUSSION

In this study, we showed that bile salts induce the expression of several *B. thetaiotaomicron* RND-efflux pump, known to be involved in the efflux of a wide range of substrates (17, 21, 30). We then demonstrated that only the deletion of one bile-induced pump composed of BT3337, BT3338, and BT3339—renamed BipABC—decreased biofilm formation and is therefore a new determinant of *B. thetaiotaomicron* biofilm formation.

In addition to BipABC, we showed that six other *B. thetaiotaomicron* RND-efflux pumps were up-regulated in the presence of bile; however, none of them played a role in biofilm formation, suggesting that these pumps could mediate other adaptative phenotypes. RND-efflux pumps are tripartite protein complexes composed of an inner pump, determining the substrate specificity, a periplasmic adapter, and a passive outer membrane channel (17). These pumps mediate the efflux of various metabolites, and some of the identified pumps could for instance mediate the efflux of bile out of the cell as a tolerance mechanism against bile cytotoxic and detergent properties, as already demonstrated for *BT2793-2795* pump in *B. thetaiotaomicron* (31) and for RND-type efflux pumps in several enteric bacteria (18, 32, 33).

Besides bile-induced stress tolerance, the observed gene expression changes in the presence of bile suggest that even at non-inhibitory concentration, bile is sensed by *B. thetaiotaomicron* and can deeply remodel its physiology. Induction of biofilm formation by bile could for instance be used as a host-derived signal to switch from planktonic to a biofilm lifestyle to stably colonize the gut or optimized food foraging in nutrient and bile-rich region of the digestive tract. We do not yet know how bile is sensed by the cell nor how it translates into mediating efflux pump expression. Interestingly, we showed that bile also modulates the transcription of some AraC-family transcriptional regulators, which are known regulators of RND-efflux pumps, with both positive and negative impact on biofilm formation (15, 18, 34, 35), and we are currently investigating their potential contribution to *B. thetaiotaomicron* biofilm formation.

By contrast with the known scaffolding role of eDNA in many bacterial species (14, 27, 28, 36), *B. thetaiotaomicron* biofilm formation in the presence of bile was previously shown to involve the degradation of eDNA by the DNase BT3563 released in the extracellular matrix (14). Here, we demonstrate that the addition of magnesium to the extracellular matrix, either through BipABC pump activity or through direct magnesium supplementation, led to lower eDNA concentration in the biofilm matrix and increased biofilm formation, confirming that excess of eDNA impairs *B. thetaiotaomicron* biofilm formation. Since many DNases require divalent cation for activity (37), we hypothesized that BipABC-dependent magnesium efflux could increase the eDNA degradation by DNases. However, we showed that extracellular DNase activity on *B. thetaiotaomicron* genomic DNA was not impaired in a *bipABC* mutant or in the presence of the pump inhibitor PAβN. Moreover, our analysis of eDNA profile showed that, even in a *∆BT3563* mutant, the addition of magnesium leads to the disappearance of the above 600 bp DNA fraction. This suggests that magnesium could reduce eDNA concentration through an unidentified DNase whose activity in not detectable in our general extracellular DNase assay (Fig. 3B). Alternatively, magnesium efflux in the biofilm matrix could limit the release of eDNA rather than impacting eDNA degradation.

One of the contributions of eDNA to biofilm architecture lies on its capacity to interact with charged components of the biofilm matrix (27, 38–40). We previously hypothesized that eDNA degradation by BT3563 could prevent or reduce electrostatic repulsion between an interbacterial grid of negatively charged eDNA molecule and negatively charged *B. thetaiotaomicron* surface and lead to denser biofilm structure (14). The high eDNA concentration associated to blocking magnesium efflux bloc would therefore increase the interbacterial distance between cells, whereas the addition of extracellular magnesium restored WT distance between bacteria and strengthened biofilm formation. However, it is yet unclear whether magnesium efflux only reduces eDNA concentration in the matrix, allowing the formation of denser biofilm, or whether it directly interacts with cells and/or eDNA in the matrix and mediates electrostatic repulsion regardless of its impact on eDNA concentration. Indeed, magnesium divalent cations are important electrostatic component in biofilms (41, 42), and the requirement for its BipABC-dependent efflux suggests that, in addition to, or independently of eDNA degradation, efflux of magnesium could neutralize the negative charge conferred by degraded or undegraded eDNA phosphate‐backbone groups (27, 28). By reducing the electrostatic repulsion forces between the matrix components, including those exposed before and after eDNA degradation, this could change the biofilm viscoelastic properties and allow closer interactions between bacteria, forming thicker and more compact biofilm structures (27).

In conclusion, our study provides evidence for a direct link between exposure to bile salts addition and factors necessary for *B. thetaiotaomicron* biofilm formation. Since biofilm formation likely provides a protective environment against environmental factors, the identification of BipABC-mediated magnesium efflux as a mechanistic link between an intestinal cue such as bile and *B. thetaiotaomicron* biofilm could lead to original strategies to foster biofilm formation by this important gut symbiont and potentially promote microbiota resilience to stress and dysbiosis events.

## MATERIALS AND METHODS

### Bacterial strains and growth conditions

*B. thetaiotaomicron* strains were grown in BHIS broth (43) supplemented with bile extract from bovine and ovine (Sigma, B8381) at 0.5%, PAßN at 25 µg/mL, magnesium at 35 mM, unless indicated otherwise, EDTA, calcium, iron, potassium, cobalt, cadmium, or zinc. Cultures were incubated at 37°C in anaerobic conditions in a C400M Ruskinn anaerobic-microaerophilic station. All media and chemicals were purchased from Sigma-Aldrich unless indicated otherwise. All experiments and genetic constructions of *B. thetaiotaomicron* were made in VPI-5482Δtdk background (44), which was developed for a two-step selection procedure of unmarked gene deletion by allelic exchange and has become the reference strain used in most studies. Therefore, VPI-5482Δtdk is referred to as wild type or VPI-5482 in this study.

### Construction of *B. thetaiotaomicron* mutants

For the generation of deletion strains by allelic exchange in *B. thetaiotaomicron*, we used the pLGB13 vector (45). Briefly, 1 kb upstream and downstream regions of the target sequence were cloned into the pLGB13 vector and transformed into the *Escherichia coli* S17 λpir strain, which was used to deliver the vector to *B. thetaiotaomicron* by conjugation. Conjugation was carried out by mixing exponentially grown cultures of the donor and the recipient strains at a 2:1 ratio and placing the mixture on BHIS agar plates at 37°C under aerobic conditions overnight. After this, bacteria were plated on selective BHIS agar supplemented with erythromycin for selection of *B. thetaiotaomicron* transconjugants that underwent the first recombination event and gentamicin to ensure the exclusion of any *E. coli* growth. Erythromycin- and gentamicin-resistant colonies of *B. thetaiotaomicron* were then subjected to a second round of selection on BHIS agar plates supplemented with anhydrotetracycline (aTC) for selection of double recombined colonies. The resulting deletion mutants were confirmed by PCR and sanger sequencing. To complement the *∆bipABC* mutant, we used the pNBU2-bla-erm vector (46), which inserts in the untranslated region of the tRNA-Ser, in which we previously cloned the constitutive promoter of the *BT1311* gene encoding the sigma factor RpoD (11). Target genes were amplified by PCR using Phusion Flash High-Fidelity PCR Master Mix from start codon to stop codon, and they were cloned in front of the BT1311 promoter by Gibson assembly. The Gibson mix was transformed in *E. coli* S17λpir, the sequence of the resulting plasmid (p*BipABC*) was verified, and *E. coli* S17λpir carrying pBipABC or pEmpty vector (= pNBU2-bla-erm) was used to transfer the plasmids into *B. thetaiotaomicron* by conjugation (see above). The final constructs were checked by PCR and sequencing.

### Growth curve

Overnight cultures of 2.5 µL were added to 200 µL BHIS that had previously been incubated in anaerobic condition overnight to remove dissolved oxygen, without or with 0.5% bile salts in Greiner flat-bottom 96-well plates. A plastic adhesive film (adhesive sealing sheet, Thermo Scientific, AB0558) was added on top of the plate inside the anaerobic station, and the plates were then incubated in a TECAN Infinite M200 Pro spectrophotometer for 24 h at 37°C. OD_600_ was measured every 30 min after a 900-s orbital shaking of 2 mm amplitude.

### Determination of minimal inhibitory concentration for vancomycin

*B. thetaiotaomicron* vancomycin E-test was performed on *Brucella* agar supplemented with hemin (5 µg/mL), vitamin K1 (1 µg/mL), and lysed horse blood [5% (vol/vol)]. To obtain a bacterial lawn, the agar dishes were covered with a soft-agar lawn composed of *Brucella* agar 0.4% supplemented with hemin (5 µg/mL), vitamin K1 (1 µg/mL), and lysed horse blood [5% (vol/vol)] and inoculated with overnight culture diluted to OD_600_ = 0.1 [final concentration 10^6^ colony-forming unit (CFU)/mL] in technical triplicates. The vancomycin E-test, purchased from bioMérieux, was placed on the agar plate and incubated at 35°C in anaerobic conditions for 48 h. Minimum inhibitory concentrations are defined by the lowest concentration of an antimicrobial that will inhibit the visible growth of a microorganism after incubation and were read directly on the graduated scale at the intersection between the inhibition ellipse and the strip (in μg/mL).

### CFU count

The CFU count was performed by a 10-times serial dilution in 200 µL of BHIS before and after the PAßN and/or bile salts exposure. A 15 µL drop of each dilution for each condition was placed on BHIS plates and incubated at 37°C in an anaerobic chamber for 48 h before the CFU count. For biofilm condition, the biofilm was harvested and centrifugated at 5,000  ×  *g* for 10  min. The pellet was weighed, then resuspended in BHIS at a 1:10 mass-volume ratio, and then the CFU count was performed as described above.

### Hoechst H33342 bisbenzimide accumulation assay

Strains were cultured overnight at 37°C and used to inoculate fresh medium that was incubated for further 5 h at 37°C. Bacterial cells were collected by centrifugation at 4,000 ×  *g* and resuspended in PBS (1 mL), as previously described (25). The optical density of all suspensions was adjusted to 0.1 at 600 nm, and aliquots (180 µL) were transferred to Greiner flat-bottom 96-well plates. Eight technical replicates of each strain were analyzed in each column. The plate was transferred to a TECAN Infinite M200 Pro spectrophotometer and incubated at 37°C, and H33342 (25 mM) was added (20 µL) to each well to give a final concentration of 2.5 mM. Fluorescence was read from the top of the wells using excitation and emission filters of 346 and 460 nm, respectively, with 5 flashes/well; readings were taken for 30 cycles with a 75 s delay between cycles and a gain multiplier of 1,460. Raw fluorescence values were analyzed using Excel (Microsoft) that included the calculation of mean values for each column and the subtraction of appropriate control blanks. Three independent experiments were performed.

### Ninety-six-well crystal violet biofilm formation assay

Overnight cultures were diluted to OD_600_ = 0.05 in 150 µL BHIS without or with supplement and inoculated in technical duplicates in polystyrene Greiner round-bottom 96-well plates. The wells at the border of the plates were filled with 200 µL of water to prevent evaporation. Incubation was done at 37°C in anaerobic conditions for 48 h. The supernatant was removed by careful pipetting, and the biofilms were fixed using 150 µL of Bouin’s solution (picric acid 0.9%, formaldehyde 9%, and acetic acid 5%, HT10132, Sigma-Aldrich) for 10 min. Then the wells were washed once with water by immersion and flicking, and the biofilm was stained with 175 µL of 1% crystal violet (V5265, Sigma-Aldrich) for 10 min. Crystal violet solution was removed by flicking, and biofilms were washed twice with water. Stained biofilms were dried then resuspended in 1:4 acetone:ethanol mix, and absorbance at 595 nm was measured using TECAN infinite M200 PRO plate reader.

### Extracellular matrix extraction and quantification

Matrix components were extracted based on previously described methods (47). Two microliter of 48 h biofilms cells grown in the presence of bile was harvested and centrifugated at 5,000  ×  *g* for 10 min. The pellet was washed twice with NaCl 0.85% and weighed, then resuspended in an extraction buffer (Tris-HCl pH 8.0; 1.5 M NaCl) at a 1:10 mass-volume ratio and incubated at 25°C for 30 min with agitation. Then, cells were removed by centrifugation at 15,000 × *g* and 25°C for 10 min, and the supernatant containing the extracted ECMs were stored at −20°C until use. The amount of DNA and proteins in the ECM was measured using a Qubit 3.0 Fluorometer (Thermo Fisher Scientific) with the Qubit dsDNA HS Assay Kit and the Qubit Protein Assay Kit, according to the manufacturer’s instructions. The concentration of polysaccharides was quantified by adding to a volume of ECM, in clean and acid-washed glass tubes, a 1:1 vol ratio of 5% phenol then 1:5 vol ration of 93% sulfuric acid. After incubation at room temperature for 10 min, 100 µL was transferred in polystyrene Greiner flat-bottom 96-well plates prior to OD_490_ measurement using a TECAN Infinite M200 Pro spectrophotometer. The polysaccharide concentration was calculated from a standard curve performed with standards of 0, 30, 60, 90, and 120 µg/mL of carbohydrate. The standards were prepared from a solution of 1 mg/mL carbohydrate composed of 50:50 of sucrose and fructose and diluted into 1 mL aliquots to obtain the above-mentioned concentrations. The eDNA profile was performed by adding 1 µL of ECM samples in an Agilent DNA chip and analyzed using the Agilent 2100 Bioanalyzer system.

### Magnesium quantification assay

Using the Magnesium Assay kit (Sigma-Aldrich, MAK026), the magnesium concentration was determined by a coupled enzyme assay that takes advantage of the specific requirement of glycerol kinase for Mg^2+^, resulting in a colorimetric (450 nm) product proportional to the magnesium present. This assay exhibits no detectable interference with Fe^2+^, Cu^2+^, Ni^2+^, Zn^2+^, Co^2+^, Ca^2+^, and Mn^2+^. Briefly, 25 µL of samples is added to 25 µL of water, into duplicate in a 96-well flat-bottom plate. In parallel, standard wells containing 0 (blank), 3, 6, 9, 12, and 15 nmole/well standards of magnesium were prepared. Then, 50 µL of the Reaction Mix was added to wells containing samples and standards. The absorbance at 450 nm (A_450_) was measured every 5 min using TECAN infinite M200 PRO plate reader in kinetic mode for 30 min at 37°C. The concentration was then calculated from a standard curve.

### Nuclease activity of the supernatant

Cultures of 48 h were centrifugated 6.5 min at 6,000 ×  *g*. Fifty microliter of supernatant was mixed with *B. thetaiotaomicron* VPI-5482 genomic DNA (800 ng/mL final concentration). Ten microliter was migrated on a 1% agarose gel and colored with ethidium bromide. The remaining 40 µL was incubated at 37°C overnight for 24 h, and then 10 µL was used to run a 1% agarose gel and colored with ethidium bromide.

### Confocal laser scanning microscopy

Biofilms were grown in 96-well plates (μclear, Greiner Bio-One). A total of 150 µL of BHIS, supplemented with 0.5% bile extract, 25 µg/mL of PAßN, or 35 mM magnesium when required, was added to each well, and the plates were incubated at 37°C, in static condition 48 h under anaerobic conditions. The unwashed biofilms were then directly stained in red with 5 µM of SYTO61 (Life Technologies; cell permeant nucleic acid dye to contrast all the bacteria). After 15 min of incubation, Z stacks of horizontal plane images were acquired in 1 µm steps using CLSM (Leica TCS SP8, INRAE MIMA2 microscopy platform) with a water 63 × immersion lens [numerical aperture (NA) = 1.2]. Two stacks of images were acquired randomly on three independent samples at 800 Hz. Fluorophores were excited, and emissions were captured as prescribed by the manufacturer. Simulated 3D fluorescence projections were generated using IMARIS 9.3 software (Bitplane). Biofilm biovolumes (µm^3^) extracted from CLSM images were analyzed with BiofilmQ (48).

### Transmission electron microscopy

For transmission electron microscopy, biofilms were grown in Falcon clear PET membrane insert for 12-well plate (ref. 353180) in 12-well plates (Greiner Bio-One). One milliliter of BHIS, supplemented with 0.5% bile extract, 25 µg/mL PAßN, or 35 mM magnesium when required, was added to each well, and the plates were incubated at 37°C, in static condition 48 h under anaerobic conditions. The unwashed biofilms were then directly fixed in a 0.07 M cacodylate buffer containing 1.3% glutaraldehyde and 0.05% ruthenium red. Samples were then washed in cacodylate-buffer and post-fixed by incubation with 1% osmium tetroxide for 1 h. Samples were then fully dehydrated in a graded series of ethanol solutions and embedded in Epon resin, which was allowed to polymerize from 37°C to 60°C. Ultra-thin sections of these blocks were obtained with an ultramicrotome. Sections were stained with 5% uranyl acetate 5% lead citrate, and observations were made with a transmission electron microscope (JEOL 1011, Tokyo, Japan).

### RNA-seq analysis

Overnight cultures were mixed with RNAprotect (Qiagen) in the anaerobic chamber to prevent RNA degradation, and bacteria were lysed using QIAGEN Proteinase K and TE buffer containing lysozyme. Total RNA was extracted using the Direct Zol kit (Zymo Cat. R2050) according to the manufacturer’s instructions and treated with DNase I from the same kit. RNA concentration, quality, and integrity from four independent replicates were checked using RNA6000 Nano chips and the Agilent 2100 Bioanalyzer system. Sequencing was performed by the Biomics platform at the Institut Pasteur. Ribosomal RNA depletion was performed using the Bacteria RiboZero kit (Illumina). From rRNA-depleted RNA, directional libraries were prepared using the TruSeq Stranded mRNA Sample preparation kit following the manufacturer’s instructions (Illumina). Libraries were checked for quality on Bioanalyzer DNA 1000 chips (Agilent). Quantification was performed with the fluorescent-based quantitation Qubit dsDNA HS Assay Kit (Thermo Fisher Scientific). Sequencing was performed as a Single Read run for 75 bp sequences on a NextSeq500 Illumina sequencer. The multiplexing level was 16 samples per lane. The RNA-seq analysis was performed with Sequana (49). In particular, we used the RNA-seq pipeline (v0.15.1, https://github.com/sequana/rnaseq) built on top of Snakemake v6.7.0 (50). The pipeline trimmed reads from adapters and low-quality bases using fastp software v0.20.1 (51), then mapped the remaining reads to the *B. thetaiotaomicron* genome (accession NC_004663.1) with bowtie2 v2.4.2 (52). Mapped reads were then quantified using featureCounts (53), and differential analysis was performed with DESeq2 v1.30.0 (54) with default parameters. Results are partially presented in Table S2 and presented in full in Table S3.

## Data Availability

The raw sequencing data for this study have been deposited in the European Nucleotide Archive (ENA) at https://www.ebi.ac.uk/biostudies/arrayexpress/studies/E-MTAB-13460 (Array Express accession number E-MTAB-13460).

## SUPPLEMENTAL MATERIAL

Supplemental material mbio.03488-23-s0001.pdf Figures S1 to S6 and Tables S1 and S2.

Table S3 mbio.03488-23-s0002.xlsx RNA-seq analysis.

ASM does not own the copyrights to Supplemental Material that may be linked to, or accessed through, an article. The authors have granted ASM a non-exclusive, world-wide license to publish the Supplemental Material files. Please contact the corresponding author directly for reuse.

## References

[1] Xu J, Gordon JI. 2003. Honor thy symbionts. Proc Natl Acad Sci U S A 100:10452–10459. doi:10.1073/pnas.1734063100 12923294 PMC193582

[2] Sonnenburg JL, Angenent LT, Gordon JI. 2004. Getting a grip on things: how do communities of bacterial symbionts become established in our intestine Nat Immunol 5:569–573. doi:10.1038/ni1079 15164016

[3] Sonnenburg JL, Xu J, Leip DD, Chen CH, Westover BP, Weatherford J, Buhler JD, Gordon JI. 2005. Glycan foraging in vivo by an intestine-adapted bacterial symbiont. Science 307:1955–1959. doi:10.1126/science.1109051 15790854

[4] Wexler HM. 2007. Bacteroides: the good, the bad, and the nitty-gritty. Clin Microbiol Rev 20:593–621. doi:10.1128/CMR.00008-07 17934076 PMC2176045

[5] Ouwerkerk JP, de Vos WM, Belzer C. 2013. Glycobiome: bacteria and mucus at the epithelial interface. Best Pract Res Clin Gastroenterol 27:25–38. doi:10.1016/j.bpg.2013.03.001 23768550

[6] Martens EC, Chiang HC, Gordon JI. 2008. Mucosal glycan foraging enhances fitness and transmission of a saccharolytic human gut bacterial symbiont. Cell Host Microbe 4:447–457. doi:10.1016/j.chom.2008.09.007 18996345 PMC2605320

[7] Motta JP, Wallace JL, Buret AG, Deraison C, Vergnolle N. 2021. Gastrointestinal biofilms in health and disease. Nat Rev Gastroenterol Hepatol 18:314–334. doi:10.1038/s41575-020-00397-y 33510461

[8] Béchon N, Ghigo JM. 2022. gut biofilms: bacteroides as model symbionts to study biofilm formation by intestinal anaerobes. FEMS Microbiol Rev 46:fuab054. doi:10.1093/femsre/fuab054 34849798

[9] Xu Q, Shoji M, Shibata S, Naito M, Sato K, Elsliger MA, Grant JC, Axelrod HL, Chiu HJ, Farr CL, Jaroszewski L, Knuth MW, Deacon AM, Godzik A, Lesley SA, Curtis MA, Nakayama K, Wilson IA. 2016. A distinct type of pilus from the human microbiome. Cell 165:690–703. doi:10.1016/j.cell.2016.03.016 27062925 PMC4842110

[10] Grondin JM, Tamura K, Déjean G, Abbott DW, Brumer H. 2017. Polysaccharide utilization loci: fueling microbial communities. J Bacteriol 199:e00860-16. doi:10.1128/JB.00860-16 28138099 PMC5512228

[11] Mihajlovic J, Bechon N, Ivanova C, Chain F, Almeida A, Langella P, Beloin C, Ghigo JM. 2019. A putative type V pilus contributes to Bacteroides thetaiotaomicron biofilm formation capacity. J Bacteriol 201:e00650-18. doi:10.1128/JB.00650-18 30833358 PMC6707919

[12] Béchon N, Mihajlovic J, Vendrell-Fernández S, Chain F, Langella P, Beloin C, Ghigo JM. 2020. Capsular polysaccharide cross-regulation modulates Bacteroides thetaiotaomicron biofilm formation. mBio 11:e00729-20. doi:10.1128/mBio.00729-20 32576670 PMC7315117

[13] Maldonado-Valderrama J, Wilde P, Macierzanka A, Mackie A. 2011. The role of bile salts in digestion. Adv Colloid Interface Sci 165:36–46. doi:10.1016/j.cis.2010.12.002 21236400

[14] Béchon N, Mihajlovic J, Lopes AA, Vendrell-Fernández S, Deschamps J, Briandet R, Sismeiro O, Martin-Verstraete I, Dupuy B, Ghigo JM. 2022. Bacteroides thetaiotaomicron uses a widespread extracellular DNase to promote bile-dependent biofilm formation. Proc Natl Acad Sci U S A 119:e2111228119. doi:10.1073/pnas.2111228119 35145026 PMC8851478

[15] Gunn JS. 2000. Mechanisms of bacterial resistance and response to bile. Microbes Infect 2:907–913. doi:10.1016/s1286-4579(00)00392-0 10962274

[16] Prouty AM, Brodsky IE, Falkow S, Gunn JS. 2004. Bile-salt-mediated induction of antimicrobial and bile resistance in Salmonella typhimurium. Microbiology (Reading) 150:775–783. doi:10.1099/mic.0.26769-0 15073288

[17] Nikaido H, Takatsuka Y. 2009. Mechanisms of RND multidrug efflux pumps. Biochim Biophys Acta 1794:769–781. doi:10.1016/j.bbapap.2008.10.004 19026770 PMC2696896

[18] Nickerson KP, Chanin RB, Sistrunk JR, Rasko DA, Fink PJ, Barry EM, Nataro JP, Faherty CS. 2017. Analysis of shigella flexneri resistance, biofilm formation, and transcriptional profile in response to bile salts. Infect Immun 85. doi:10.1128/IAI.01067-16 PMC5442615 28348056

[19] Tseng TT, Gratwick KS, Kollman J, Park D, Nies DH, Goffeau A, Saier MH. 1999. The RND permease superfamily: an ancient, ubiquitous and diverse family that includes human disease and development proteins. J Mol Microbiol Biotechnol 1:107–125. 10941792

[20] Lin J, Cagliero C, Guo B, Barton YW, Maurel MC, Payot S, Zhang Q. 2005. Bile salts modulate expression of the CmeABC multidrug efflux pump in Campylobacter jejuni. J Bacteriol 187:7417–7424. doi:10.1128/JB.187.21.7417-7424.2005 16237025 PMC1272998

[21] Henderson PJF, Maher C, Elbourne LDH, Eijkelkamp BA, Paulsen IT, Hassan KA. 2021. “Physiological functions of bacterial "multidrug" efflux pumps”. Chem Rev 121:5417–5478. doi:10.1021/acs.chemrev.0c01226 33761243

[22] Lomovskaya O, Warren MS, Lee A, Galazzo J, Fronko R, Lee M, Blais J, Cho D, Chamberland S, Renau T, Leger R, Hecker S, Watkins W, Hoshino K, Ishida H, Lee VJ. 2001. Identification and characterization of inhibitors of multidrug resistance efflux pumps in Pseudomonas aeruginosa: novel agents for combination therapy. Antimicrob Agents Chemother 45:105–116. doi:10.1128/AAC.45.1.105-116.2001 11120952 PMC90247

[23] Marquez B. 2005. Bacterial efflux systems and efflux pumps inhibitors. Biochimie 87:1137–1147. doi:10.1016/j.biochi.2005.04.012 15951096

[24] Misra R, Morrison KD, Cho HJ, Khuu T. 2015. Importance of real-time assays to distinguish multidrug efflux pump-inhibiting and outer membrane-destabilizing activities in Escherichia coli. J Bacteriol 197:2479–2488. doi:10.1128/JB.02456-14 25962916 PMC4518837

[25] Coldham NG, Webber M, Woodward MJ, Piddock LJV. 2010. A 96-well plate fluorescence assay for assessment of cellular permeability and active efflux in Salmonella enterica serovar typhimurium and Escherichia coli. J Antimicrob Chemother 65:1655–1663. doi:10.1093/jac/dkq169 20513705

[26] Elbourne LDH, Tetu SG, Hassan KA, Paulsen IT. 2017. TransportDB 2.0: a database for exploring membrane transporters in sequenced genomes from all domains of life. Nucleic Acids Res 45:D320–D324. doi:10.1093/nar/gkw1068 27899676 PMC5210551

[27] Flemming H-C, van Hullebusch ED, Neu TR, Nielsen PH, Seviour T, Stoodley P, Wingender J, Wuertz S. 2023. The biofilm matrix: multitasking in a shared space. Nat Rev Microbiol 21:70–86. doi:10.1038/s41579-022-00791-0 36127518

[28] Okshevsky M, Meyer RL. 2015. The role of extracellular DNA in the establishment, maintenance and perpetuation of bacterial biofilms. Crit Rev Microbiol 41:341–352. doi:10.3109/1040841X.2013.841639 24303798

[29] Beloin C, Valle J, Latour-Lambert P, Faure P, Kzreminski M, Balestrino D, Haagensen JAJ, Molin S, Prensier G, Arbeille B, Ghigo J-M. 2004. Global impact of mature biofilm lifestyle on Escherichia coli K-12 gene expression. Mol Microbiol 51:659–674. doi:10.1046/j.1365-2958.2003.03865.x 14731270

[30] Alav I, Kobylka J, Kuth MS, Pos KM, Picard M, Blair JMA, Bavro VN. 2021. Structure, assembly, and function of tripartite efflux and type 1 secretion systems in gram-negative bacteria. Chem Rev 121:5479–5596. doi:10.1021/acs.chemrev.1c00055 33909410 PMC8277102

[31] Liu H, Shiver AL, Price MN, Carlson HK, Trotter VV, Chen Y, Escalante V, Ray J, Hern KE, Petzold CJ, Turnbaugh PJ, Huang KC, Arkin AP, Deutschbauer AM. 2021. Functional genetics of human gut commensal Bacteroides thetaiotaomicron reveals metabolic requirements for growth across environments. Cell Reports 34:108789. doi:10.1016/j.celrep.2021.108789 33657378 PMC8121099

[32] Piddock LJV. 2006. Multidrug-resistance efflux pumps - not just for resistance. Nat Rev Microbiol 4:629–636. doi:10.1038/nrmicro1464 16845433

[33] Sistrunk JR, Nickerson KP, Chanin RB, Rasko DA, Faherty CS. 2016. Survival of the fittest: how bacterial pathogens utilize bile to enhance infection. Clin Microbiol Rev 29:819–836. doi:10.1128/CMR.00031-16 27464994 PMC5010752

[34] Duval V, Lister IM. 2013. MarA, SoxS and Rob of Escherichia coli - global regulators of multidrug resistance, virulence and stress response. Int J Biotechnol Wellness Ind 2:101–124. doi:10.6000/1927-3037.2013.02.03.2 24860636 PMC4031692

[35] Kettles RA, Tschowri N, Lyons KJ, Sharma P, Hengge R, Webber MA, Grainger DC. 2019. The Escherichia coli MarA protein regulates the ycgZ-ymgABC operon to inhibit biofilm formation. Mol Microbiol 112:1609–1625. doi:10.1111/mmi.14386 31518447 PMC6900184

[36] Whitchurch CB, Tolker-Nielsen T, Ragas PC, Mattick JS. 2002. Extracellular DNA required for bacterial biofilm formation. Science 295:1487. doi:10.1126/science.295.5559.1487 11859186

[37] Guéroult M, Picot D, Abi-Ghanem J, Hartmann B, Baaden M. 2010. How cations can assist DNase I in DNA binding and hydrolysis. PLoS Comput Biol 6:e1001000. doi:10.1371/journal.pcbi.1001000 21124947 PMC2987838

[38] Dengler V, Foulston L, DeFrancesco AS, Losick R. 2015. An electrostatic net model for the role of extracellular DNA in biofilm formation by Staphylococcus aureus. J Bacteriol 197:3779–3787. doi:10.1128/JB.00726-15 26416831 PMC4652055

[39] Kavanaugh JS, Flack CE, Lister J, Ricker EB, Ibberson CB, Jenul C, Moormeier DE, Delmain EA, Bayles KW, Horswill AR. 2019. Identification of extracellular DNA-binding proteins in the biofilm matrix. mBio 10:e01137-19. doi:10.1128/mBio.01137-19 31239382 PMC6593408

[40] Campoccia D, Montanaro L, Arciola CR. 2021. Extracellular DNA (eDNA). a major ubiquitous element of the bacterial biofilm architecture. Int J Mol Sci 22:9100. doi:10.3390/ijms22169100 34445806 PMC8396552

[41] Oknin H, Steinberg D, Shemesh M. 2015. Magnesium ions mitigate biofilm formation of Bacillus species via downregulation of matrix genes expression. Front Microbiol 6:907. doi:10.3389/fmicb.2015.00907 26441856 PMC4561805

[42] Wang T, Flint S, Palmer J. 2019. Magnesium and calcium ions: roles in bacterial cell attachment and biofilm structure maturation. Biofouling 35:959–974. doi:10.1080/08927014.2019.1674811 31687841

[43] Bacic MK, Smith CJ. 2008. Laboratory maintenance and cultivation of Bacteroides species. Curr Protoc Microbiol Chapter 13:Unit 13C.1. doi:10.1002/9780471729259.mc13c01s9 PMC3836205 18770533

[44] Koropatkin NM, Martens EC, Gordon JI, Smith TJ. 2008. Starch catabolism by a prominent human gut symbiont is directed by the recognition of amylose helices. Structure 16:1105–1115. doi:10.1016/j.str.2008.03.017 18611383 PMC2563962

[45] García-Bayona L, Comstock LE. 2019. Streamlined genetic manipulation of diverse Bacteroides and Parabacteroides isolates from the human gut microbiota. mBio 10:e01762-19. doi:10.1128/mBio.01762-19 31409684 PMC6692515

[46] Wang J, Shoemaker NB, Wang GR, Salyers AA. 2000. Characterization of a Bacteroides mobilizable transposon, NBU2, which carries a functional Lincomycin resistance gene. J Bacteriol 182:3559–3571. doi:10.1128/JB.182.12.3559-3571.2000 10852890 PMC101958

[47] Chiba A, Sugimoto S, Sato F, Hori S, Mizunoe Y. 2015. A refined technique for extraction of extracellular matrices from bacterial biofilms and its applicability. Microb Biotechnol 8:392–403. doi:10.1111/1751-7915.12155 25154775 PMC4408173

[48] Hartmann R, Jeckel H, Jelli E, Singh PK, Vaidya S, Bayer M, Rode DKH, Vidakovic L, Díaz-Pascual F, Fong JCN, Dragoš A, Lamprecht O, Thöming JG, Netter N, Häussler S, Nadell CD, Sourjik V, Kovács ÁT, Yildiz FH, Drescher K. 2021. Quantitative image analysis of microbial communities with Biofilmq. Nat Microbiol 6:151–156. doi:10.1038/s41564-020-00817-4 33398098 PMC7840502

[49] Cokelaer T, Desvillechabrol D, Legendre R, Cardon M. 2017. Sequana': a set of snakemake NGS pipelines. JOSS 2:352. doi:10.21105/joss.00352

[50] Köster J, Rahmann S. 2012. Snakemake--a scalable bioinformatics workflow engine. Bioinformatics 28:2520–2522. doi:10.1093/bioinformatics/bts480 22908215

[51] Chen S, Zhou Y, Chen Y, Gu J. 2018. Fastp: an ultra-fast all-in-one FASTQ preprocessor. Bioinformatics 34:i884–i890. doi:10.1093/bioinformatics/bty560 30423086 PMC6129281

[52] Langmead B, Salzberg SL. 2012. Fast gapped-read alignment with bowtie 2. Nat Methods 9:357–359. doi:10.1038/nmeth.1923 22388286 PMC3322381

[53] Liao Y, Smyth GK, Shi W. 2014. featureCounts: an efficient general purpose program for assigning sequence reads to genomic features. Bioinformatics 30:923–930. doi:10.1093/bioinformatics/btt656 24227677

[54] Love MI, Huber W, Anders S. 2014. Moderated estimation of fold change and dispersion for RNA-seq data with DESeq2. Genome Biol 15:550. doi:10.1186/s13059-014-0550-8 25516281 PMC4302049

