# Supplementary material for: Bile-induced biofilm formation in Bacteroides thetaiotaomicron requires magnesium efflux by an RND pump
Source: mBio. 2024 Mar 27;15(5):e03488-23. doi: 10.1128/mbio.03488-23 (PMC11078008; doi:10.1128/mbio.03488-23)
Supplement: Supplemental material — Figures S1 to S6 and Tables S1 and S2. [file mbio.03488-23-s0001.pdf]

SUPPLEMENTARY MATERIALS

Bile-induced biofilm formation in *Bacteroides thetaiotaomicron* requires magnesium efflux by an RND pump

Anne-Aurélie LOPES, Sol VENDRELL-FERNÁNDEZ, Julien DESCHAMPS, Sonia GEORGEAULT, Thomas COKELAER[5,6], Romain BRIANDET[3] and Jean-Marc GHIGO[1*]

Supporting Figures S1 to S6

Supporting Tables S1, S2 (supporting table S3 is provided as an excel spreadsheet uploaded separately as a dataset)

SUPPLEMENTARY TABLES

Supplementary Table S1: PAßN does not permeabilize the outer membrane. Vancomycin susceptibility testing was performed using E-test on VPI-5482 without and with 25 and 50 µg/mL of phenylalanine-arginine ß-naphthylamide (PAßN). Vancomycin is a large antibiotic that is normally incapable of crossing the outer membrane of Gram-negative bacteria without permeabilization of this membrane. The Minimal inhibitory Concentration (MIC) was confirmed by 3 independent experiments for each condition.

	PAßN (µg/mL)		
	0	25	50
MIC to vancomycin (µg/mL)	>256	>256	>256

Supplementary Table S2: RNA-sequencing results comparing gene expression for the 21 *B. thetaiotaomicron* RND-type efflux operons with and without 0.5% bile salts.

RND-type efflux pump operons	Genes	Compared log2 fold expression change with and without bile	Adjusted p-value
**BT4693-4695**	BT4693	-0.219	0.71
	**BT4694**	-0.124	0.73
	BT4695	-0.050	0.92
**BT3968-3969**	BT3968	-0.887	0.24
	**BT3969**	0.257	0.77
**BT3337-3339** *	BT3337	2.615	<0.001
	**BT3338**	2.847	$8.105e^{-11}$
	BT3339	2.301	$6.894e^{-22}$
**BT2940-2942**	BT2940	-0.164	0.88
	**BT2941**	-0.350	0.63
	BT2942	-0.151	0.84
**BT2835** *	**BT2835**	1.716	0.0005
**BT2793-2795** *	BT2793	7.517	$7.367e^{-49}$
	**BT2794**	7.550	$1.187e^{-68}$
	BT2795	7.644	$2.489e^{-98}$
**BT2686-2688** *	**BT2686**	3.546	$1.082e^{-34}$
	BT2687	3.270	$9.385e^{-28}$
	BT2688	2.851	$7.322e^{-79}$
**BT2251-2253**	BT2251	-0.707	0.14
	**BT2252**	0.261	0.42
	BT2253	-0.119	0.46
**BT2117-2119** *	BT2117	2.575	$1.40e^{-6}$
	**BT2118**	2.595	0.00040
	BT2119	1.809	0.0096
**BT2038-2040**	BT2038	0.436	0.28
	**BT2039**	-0.023	0.97
	BT2040	-1.074	0.04
**BT1965-1967** *	BT1965	1.965	0.009
	**BT1966**	2.637	0.00005
	BT1967	3.142	0.021
**BT1693-1695**	BT1693	-0.930	0.17
	**BT1694**	-0.258	0.79
	BT1695	-0.771	0.16
**BT1465-1468**	BT1465	0.019	0.96
	BT1466	-0.286	0.61
	**BT1467**	-1.822	0.02
	BT1468	-1.398	0.30
**BT1267-1269**	BT1267	-0.341	0.67
	**BT1268**	0.010	0.99
	BT1269	-0.999	0.58
**BT0884-0886**	BT0884	-0.532	0.55
	BT0885	-0.265	0.71
	**BT0886**	-0.419	0.47
**BT0678-0680**	BT0678	-0.166	0.87
	BT0679	0.235	0.82
	**BT0680**	-0.044	0.97
**BT0669-0672**	BT0669	-1.017	0.05
	**BT0670**	0.070	0.91
	BT0671	0.939	0.39
	**BT0672**	-0.254	0.85
**BT0304-0306**	BT0304	-1.267	0.0003
	**BT0305**	-0.2794	0.69
	BT0306	-0.308	0.63
**BT0297-0300** *	BT0297	2.139	0.06
	BT0298	2.799	0.009
	**BT0299**	1.202	0.109
	**BT0300**	2.0378	0.065

* *B. thetaiotaomicron* VPI-5482 operon deleted in this study.
Gene name **in bold** in the gene column correspond to the RND-type efflux inner pump gene.

Supporting Table S3 (provided as an excel spreadsheet dataset). RNAseq analysis: table of A. all genes, B. upregulated, C. downregulated genes in presence of 0.5% bile, D. COG functional categories enrichment.

SUPPLEMENTARY FIGURES

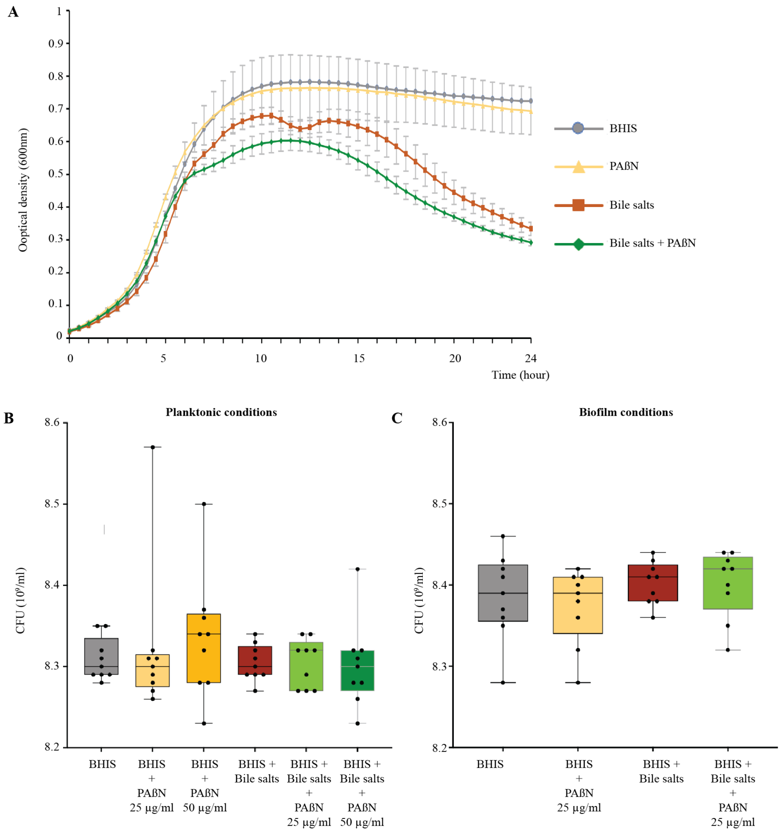

Figure S1: PAßN does not affect growth nor viability. A. 24 hours growth curve of VPI-5482 in BHIS without or with 25µg/mL of PAßN and/or 0.5% bile salts (BS) in 96-well plate. Mean of 6 biological replicates, error bars represent standard deviation to the mean (SEM). **B.** Quantification of cells in overnight planktonic cultures of VPI-5482 grown in BHIS with or without phenylalanine-arginine ß-naphthylamide (PAßN) at 25 or 50 µg/mL and/or 0.5% bile salts (BS). Min-max boxplot of 9 biological replicates for each condition. **C.** Quantification of cells in biofilms of VPI-5482 grown in BHIS with or without phenylalanine-arginine ß-naphthylamide (PAßN) at 25 µg/mL and/or 0.5% bile salts (BS). Min-max boxplot of 9 biological replicates for each condition. Statistics correspond to an unpaired, nonparametric Mann–Whitney *U* test. No significant difference was detected (p-value > 0.05).

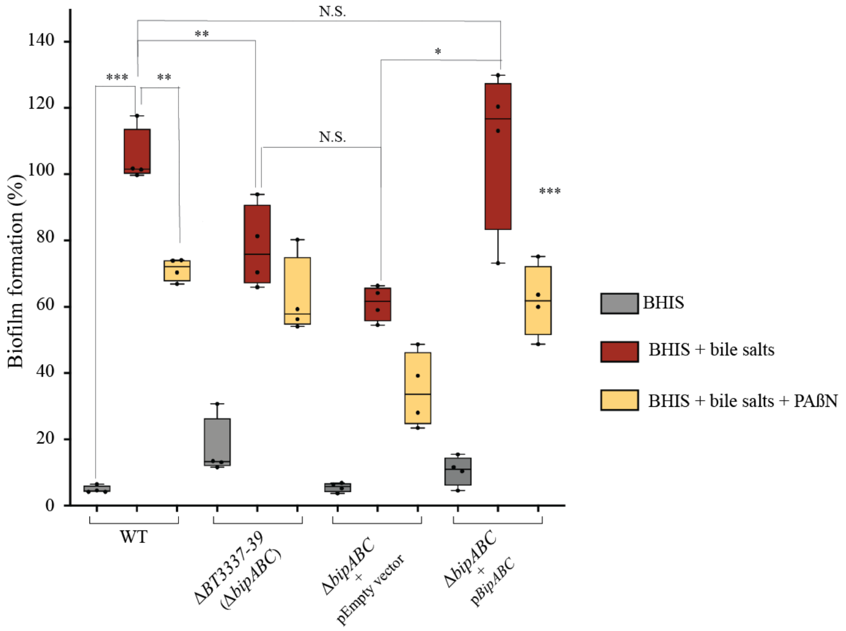

Supplementary Figure S2: Complementation of the biofilm formation defect of the Δ*bipABC* mutant. 96-well plate crystal violet biofilm assay after 48h growth in BHIS in the absence and presence of 0.5% bile salts (BS) with or without 25µg/mL of PAßN. Mean of WT in BHIS with 0.5% BS was adjusted to 100%. Min-max boxplot of 4 biological replicates, each of them being the mean of 6 technical replicates per condition. *** p-value<0.0005; NS: non-significant compared to WT in BHIS with BS. Statistics correspond to an unpaired, nonparametric Mann–Whitney *U* test.

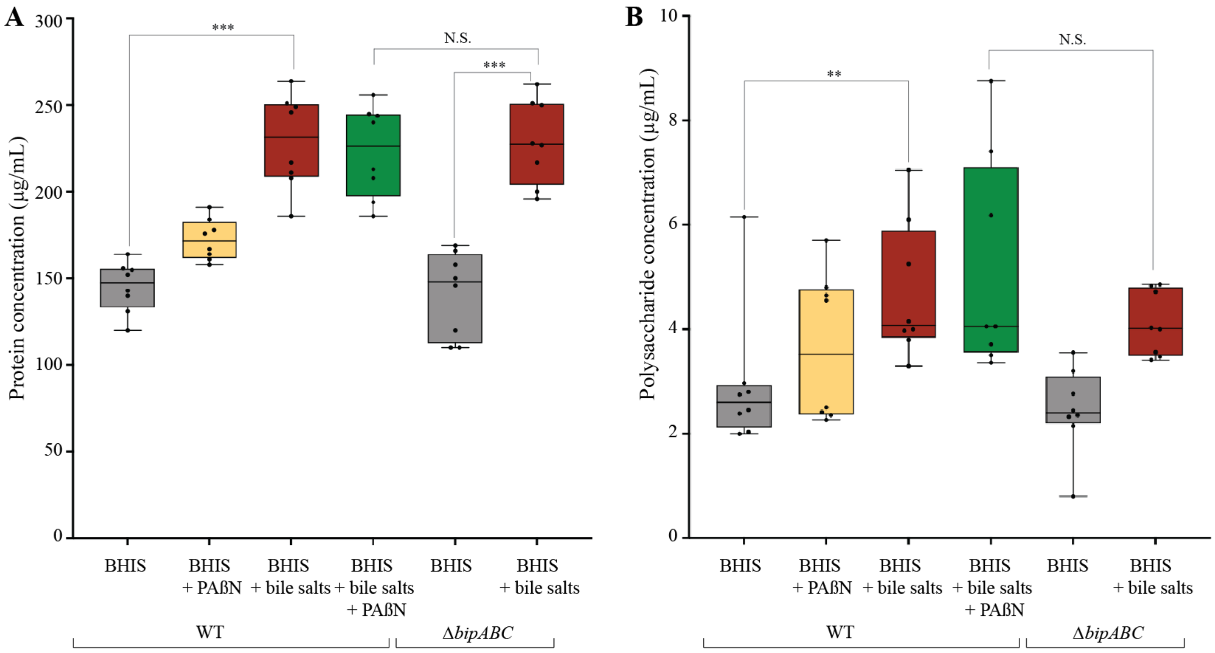

Supplementary Figure S3: Impact of impaired efflux on extracellular matrix protein and polysaccharide composition. Concentration of proteins (**A**) and polysaccharides (**B**) in the ECM of *B. thetaiotaomicron* VPI-5482 WT or its corresponding Δ*bipABC* mutant without and with 0.5% bile salts (BS) or 25 µg/mL of PAßN. Min-max boxplot of 8 biological replicates for each condition. ** p-value<0.005, *** p-value<0.0005. N.S.: non-significant. Statistics correspond to an unpaired, nonparametric Mann–Whitney *U* test.

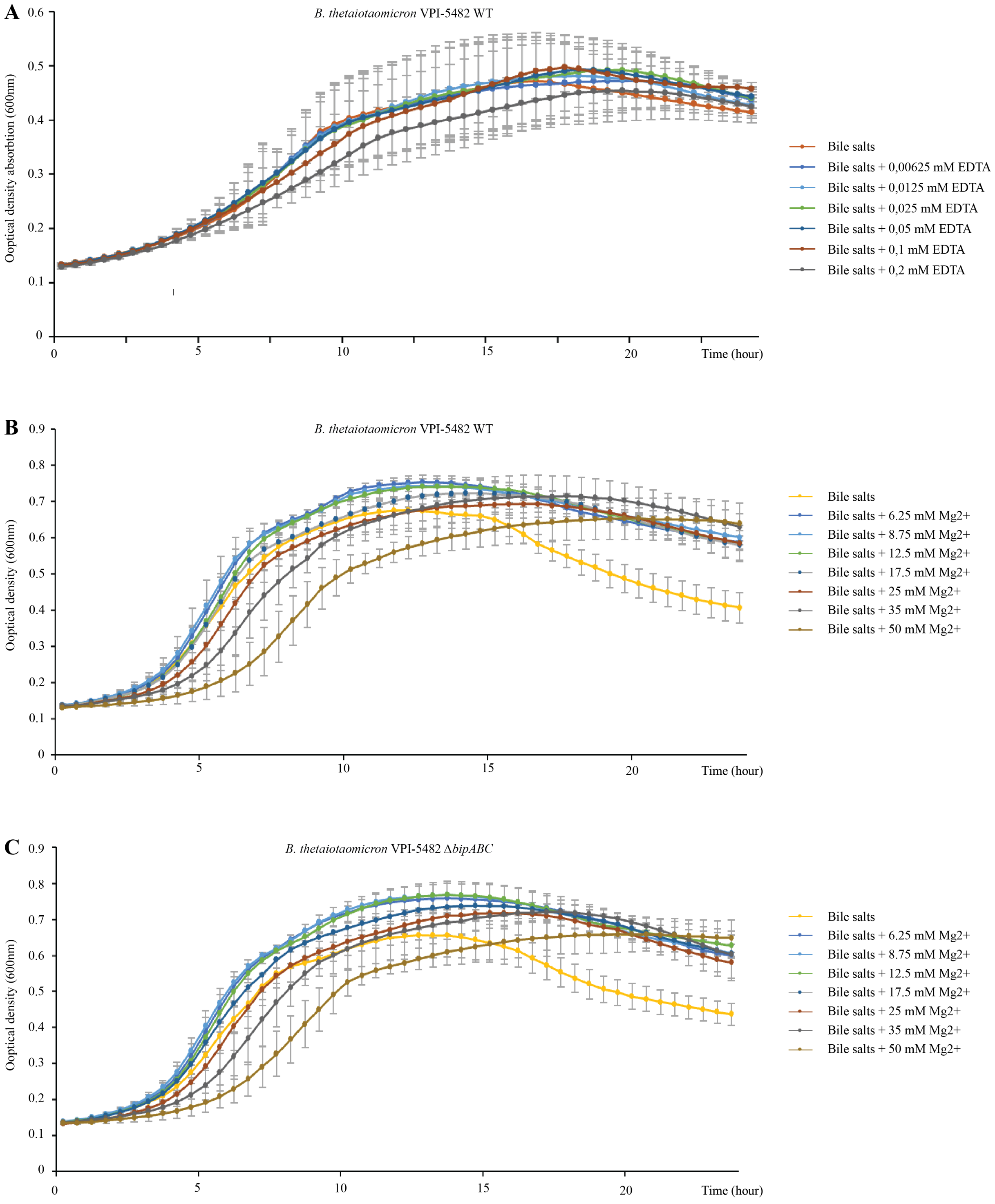

Supplementary Figure S4: Non-toxicity of EDTA and Magnesium on the strains used. A. 24 hours growth curve of *B. thetaiotaomicron* VPI-5482 in BHIS with 0.5% bile salts and different concentrations of EDTA from 0 to 0.2 mM in 96-well plate. **B and C.** 24 hours growth curve in BHIS with 0.5% bile salts and different concentrations of Magnesium from 0 to 50 mM in 96-well plate in *B. thetaiotaomicron* VPI-5482 WT (**B**) and Δ*bipABC* (**C**). Mean of 4 biological replicates, error bars represent standard deviation to the mean (SEM).

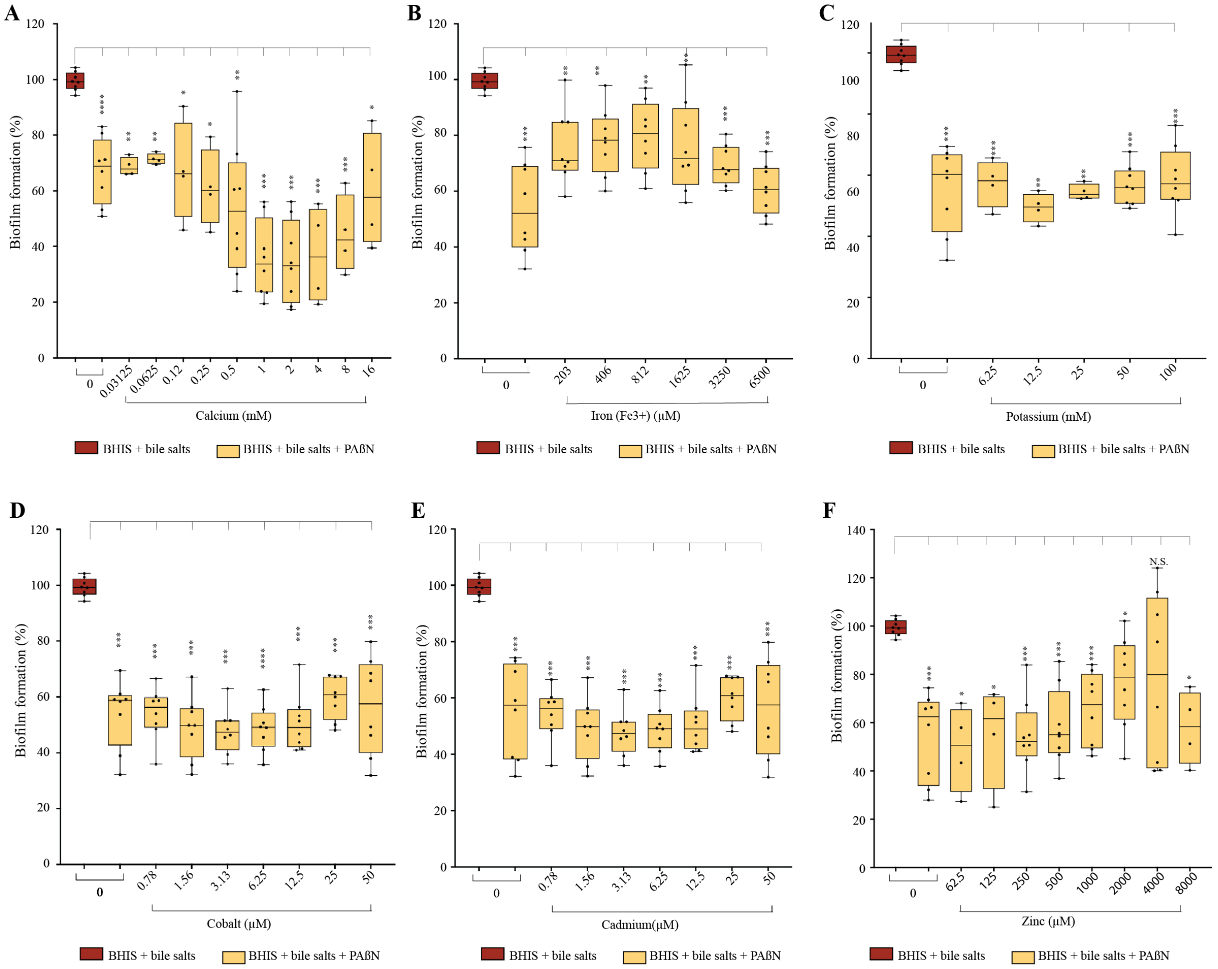

Supplementary figure S5: Addition of cations other than magnesium does not restore biofilm formation of VPI-5482 with PAßN. 96-well plate crystal violet biofilm assay after 48h growth in BHIS of VPI-5482 in the presence of 0.5% bile salts (BS), without or with 25µg/mL of PAßN and different non-toxic concentrations of calcium (**A**), iron (**B**), potassium (**C**), cobalt (**D**), cadmium (**E**) and zinc (**F**). Mean of WT in BHIS with 0.5% BS was adjusted to 100%. Min-max boxplot of 8 biological replicates for each condition. * p-value<0.05; ** p-value<0.005; *** p-value<0.0005; **** p-value<0.00005 compared to WT in BHIS with BS. N.S.: non-significant. Statistics correspond to an unpaired, nonparametric Mann–Whitney *U* test.

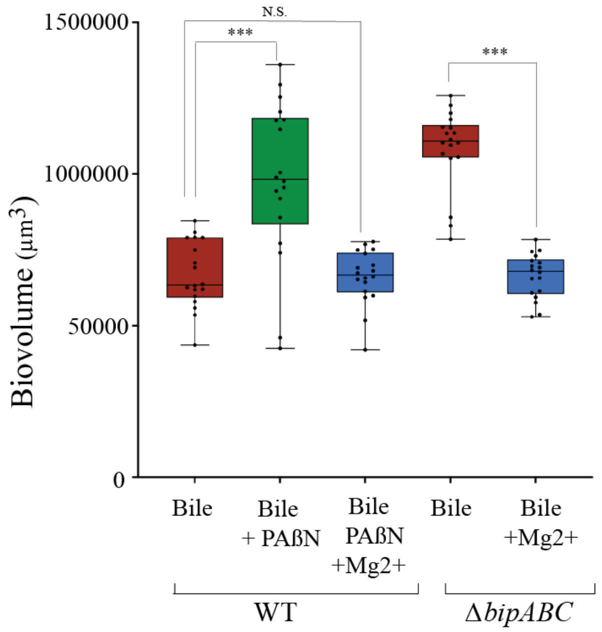

Supplementary figure S6: Increased biovolume upon addition of PAßN and deletion of the BipABC RND pump is complemented by supplementation with magnesium. Biofilm biovolumes (μm^3) extracted from CLSM images were analyzed with BiofilmQ. Each value is a mean of 18 values obtained from 3 independent experiments. Statistics correspond to an unpaired, nonparametric Mann–Whitney *U* test.
